# Impact of Toolpath Pitch Distance on Cutting Tool Nose Radius Deviation and Surface Quality of AISI D3 Steel Using Precision Measurement Techniques

**DOI:** 10.3390/ma17184519

**Published:** 2024-09-14

**Authors:** Santhakumar Jayakumar, Sathish Kannan, U. Mohammed Iqbal

**Affiliations:** 1 Department of Mechanical Engineering, Faculty of Engineering and Technology, SRM Institute of Science and Technology, Kattankulathur Campus, Chennai 603203, Tamil Nadu, India; santhakj@srmist.edu.in; 2 Department of Mechanical Engineering, Amity University, Dubai 345019, United Arab Emirates; skannan@amityuniversity.ae

**Keywords:** trochoidal toolpath, nose radius deviation, surface roughness, response surface methodology, desirability function analysis

## Abstract

The selection of the right tool path trajectory and the corresponding machining parameters for end milling is a challenge in mold and die industries. Subsequently, the selection of appropriate tool path parameters can reduce overall machining time, improve the surface finish of the workpiece, extend tool life, reduce overall cost, and improve productivity. This work aims to establish the performance of end milling process parameters and the impact of trochoidal toolpath parameters on the surface finish of AISI D3 steel. It especially focuses on the effect of the tool tip nose radius deviation on the surface quality using precision measurement techniques. The experimental design was carried out in a systematic manner using a face-centered central composite design (FCCD) within the framework of response surface methodology (RSM). Twenty different experiment trials were conducted by changing the independent variables, such as cutting speed, feed rate, and trochoidal pitch distance. The main effects and the interactions of these parameters were determined using analysis of variance (ANOVA). The optimal conditions were identified using a multiple objective optimization method based on desirability function analysis (DFA). The developed empirical models showed statistical significance with the best process parameters, which include a feed rate of 0.05 m/tooth, a trochoidal pitch distance of 1.8 mm, and a cutting speed of 78 m/min. Further, as the trochoidal pitch distance increased, variations in the tool tip cutting edge were observed on the machined surface due to peeling off of the coating layer. The flaws on the tool tip, which alter the edge micro-geometry after machining, resulted in up to 33.83% variation in the initial nose radius. Deviations of 4.25% and 5.31% were noted between actual and predicted values of surface roughness and the nose radius, respectively.

## 1. Introduction

Microdamage, chipped edges, surface defects, and flank wear at the tips of cutting tools during machining harm the tool’s life and the quality of the surface that has been machined, which in turn affects the mechanical properties of the tool [1]. Many of these problems have been investigated in studies in the literature on the geometry of the cutting tool tip and edge flutes, as well as input parameters such as feed rate and cutting speed on the surface finish of the machined workpieces [2]. The geometry of cutting tool, which includes rake angle, clearance angle, and flute design, reduces wear and enhances the machining performance as per the study [3]. According to the research it is evident that varying the feed rate, depth of cut, and cutting speed may enhance surface finish and tool life [4].

End milling is one of the most popular processes applied in the creation of various parts and products, including dies and molds. This process may use different linear or non-linear tool path patterns. High-speed machining using conventional tool paths results in increased cutting forces, machining vibrations, and tool wear and reduced accuracy [5]. To overcome these consequences, the latest research has been directed toward trochoidal milling, which incorporates circular and linear movements to reduce the shocks on dynamic cutting forces resulting from continuous variations in the radial depth of the cut. Trochoidal milling is mostly used when cutting narrow slots and other complicated pockets [6]. Milling with trochoidal movement is especially useful for cutting narrow slots and forming complex cavities on the workpiece. Nevertheless, high trochoidal toolpath parameters can increase tool wear and cutting force. It deteriorates the surface quality, and a small step parameter can hinder machining performance. Thus, assessing and forecasting the influence of toolpath parameters are vital for enhancing machining performance during trochoidal milling. Some of the recent commercial CAM software packages have integrated functions for trochoidal machining, and there are many studies in the literature on the methods of trochoidal machining [7].

Waszczuk et al. [8] employed trochoidal tool paths to assess the surface of groove walls and compared the results to conventional milling. It can be observed from the measurements that the tool path has a relationship with groove wall roughness and waviness. Waviness is more influenced by the path type as compared to the surface roughness. This is because the type of tool path trajectory that must be followed by the tool is more critical to achieving a good surface finish than the technical parameters that define the tool. Liu et al. [9] examined whether undeformed chip thickness affects chip formation and tool flank tear during dry trochoidal milling of Ti-6Al-4V alloy. A wear model is constructed taking into consideration the MRR and the radial depth of cut. Using this model, the optimal parameter settings were chosen to minimize tool wear and enhance machining efficiency.

Pleta et al. (2019) studied the parameters of trochoidal milling of Inconel 718 with an emphasis on the minimization of cutting forces and tool wear and analyzed the impact of these parameters on the depth of the machining-affected zone. These studies reveal that proper selection of parameters will greatly improve the machining rate and surface finish [10]. Yan et al. (2017) focused on stability and step optimization in trochoidal milling, where a method for the step size in the trochoidal path is discussed, which is important for maintaining stability as well as minimizing vibrations during the milling operation. This work shows that step optimization plays a crucial role in improving the machining performance [11].

Rauch et al. (2009) described the enhancements in the generation and application of trochoidal tool paths with the help of process constraints modeling. They present procedures to determine the maximum radial depth of cut and the most appropriate tool path parameters so that the performance may be improved when milling pockets [12]. In their study, Zhang et al. (2014) predicted cutting forces in trochoidal milling with a focus on the radial depth of cut, which is a model that can help in the determination of the relationship between cutting forces and milling parameters with the aim of identifying ways of improving the machining process [13].

In their study, Ibaraki et al. (2010) examined how to avoid the critical cutting regions using trochoidal grooving and focus on the application of trochoidal milling in dealing with critical cutting areas, which is useful in enhancing the performance of the milling operation [14]. Otkur and Lazoglu (2007) provided an extensive review of trochoidal milling, explaining the benefits of this approach over conventional milling techniques and exploring how trochoidal milling functions in different milling operations to set the stage for future studies in this field [15]. Finally, Shixiong et al. (2016) assessed trochoidal machining for high-speed pocket milling and stress the importance of path geometry optimization for improved tool life and machining performance. Their results are useful to improve the knowledge of how trochoidal paths can be efficiently employed in intricate milling processes [16].

Pleta et al. [17] employed the trochoidal milling technique to create a slot in a titanium alloy. Their goal was to optimize the machining time and surface quality as the parameters used for the output. Based on the research findings, it was determined that trochoidal milling is an ideal choice for slot milling applications due to its superior surface finish compared to alternative tool paths. In addition, the tool path strategy demonstrated reduced chip generation and improved chip evacuation capabilities.

Patil et al. [18] investigated the cutting forces of the trochoidal tool path strategy on a nickel-based superalloy. The experiment was performed on different metals under identical circumstances, which led to the conclusion that the cutting forces were caused by flank wear in the tool. The conventional approaches to parameter selection are tedious and time-consuming processes [19]. Therefore, the development of a prediction model and multi-objective optimization are required to enhance the output quality and performance of the machined product in end milling.

RSM was often utilized along with desirability functions for predicting optimal outcomes [20]. The study described in reference [21] developed an empirical system modeling for predicting overcut in machining using the FCCD method. The empirical model was then compared to the RSM model. The model has a strong correlation with the experimental data and was compared to the response surface methodology (RSM) model. The DFA approach is widely used in both business and academics to optimize multiple answers [22]. Jeyakumar et al. [23] examined the impact of various factors, such as the depth of cut, nose radius, spindle speed, and feed, on cutting force during the Al6061/SiC composite milling process. They employed RSM to analyze their findings. Bhardwaj et al. [24] developed a model to calculate the roughness of EN 353 steel during milling. Factors like speed, feed, depth of cut, and nose radius were considered. Surface roughness during milling was evaluated in this study. RSM was used to generate mathematical models, and genetic algorithms were used to optimize them. Prajina et al. [25] investigate the use of RSM to achieve an increased material removal rate, reduced surface roughness, and decreased force in CNC end milling processes. A second-order quadratic model is established, considering cutting parameters, such as angle, feed rate, depth of cut, and spindle speed, and output characteristics, including modeling forces, surface roughness, and machining time. The variables were determined using ANOVA. Multi-response optimization and the response surface approach were used, in conjunction with the produced models, to obtain the ideal machining settings. Iqbal et al. [26] performed RSM to estimate surface roughness and dish angle for slot milling operations using D3 steel. The process employed loop diameter as a variable, and ANOVA was used to determine the parameters. Abou-El-Hossein et al. [27] suggested both primary and secondary mathematical representations for cutting force during end milling on modified AISI P20. They used RSM to investigate the significant interactions among the feed rate, axial depth of cut, and step-over variables.

Despite extensive research on trochoidal milling and its various aspects, such as the impact of cutting parameters on tool longevity and workpiece surface quality, there has been limited investigations on the correlation between the trochoidal pitch distance and surface finish as well as tool performance. For instance, although trochoidal milling is capable of decreasing surface roughness and enhancing tool life over conventional techniques, the effect of pitch distance on surface roughness and tool tip nose radius deviation has not been well described.

Additionally, most of the previous works investigate individual factors, including feed rate and cutting speed, without taking into account the interactions with the trochoidal pitch distance and the resulting output quality measures. This also shows that the predictive modeling of these parameters concerning surface texture and tool geometry has not been well researched. The current methodologies mostly use conventional optimization techniques, which might not effectively address the dynamics of the machining process. Therefore, the present research intends to address this research gap through the systematic investigation of the effects of trochoidal pitch distance and tool tip nose radius on surface roughness and nose radius deviation with the help of advanced multi-objective optimization techniques, such as the desirability function approach and response surface methodology (RSM). This approach not only addresses the gaps in the literature but also gives a better picture of the factors affecting the machining performance in AISI D3 cold work tool steels.

Through the inclusion of trochoidal pitch distance as an input variable and tool tip nose radius deviation as the output variable in the analysis, this study aims at increasing the predictability of machining performance in end milling operations with the ultimate goal of improving process performance and product quality. The analysis of the current state of the research makes it possible to identify the need for the proposed study and its potential to contribute to the development of the precision machining field.

## 2. Materials and Methods

AISI D3 cold-worked tool steel obtained from Tradewell Ferromet Private Limited, Mumbai, India was used for this investigation. The details of the chemical composition of the steel based on weight percent and the mechanical properties of the material are shown in Table 1 and Table 2, respectively. Other materials that have characteristics similar to AISI D3 steel include AISI D2 tool steel with a hardness of 55–62 HRC, AISI D6 tool steel with a hardness of 58–64 HRC, and AISI A2 tool steel with a hardness of 58–62 HRC. These alternatives offer almost comparable levels of wear resistance, hardness, and toughness, making it easier for the manufacturers to determine the most appropriate material for their machining requirements. Its impressive deep-hardening capabilities and exceptional wear resistance from gliding contact with other metals make it a preferred option for cold-forming dies and mold manufacturing in the automotive and aerospace industries. The end milling operation was carried out on a three-axis VMC- BFW Gaurav machine (Bharat Fritz Werner Limited, Bengaluru, India), which is equipped with a Sinumerik 828D Siemens basic controller. Modules arranged along the X, Y, and Z axes provide a 450 mm × 350 mm × 350 mm workspace. It features a maximum spindle speed of 8000 rpm, a 10,000 mm/min feed rate, and a 3 kW spindle motor power. The machine’s repeatability accuracy is ±0.003 mm, and its positioning precision is ±0.005 mm. All the test trials were conducted under dry cutting conditions with a slot of 150 mm in length and 50 × 50 mm in cross-section. Table 3 lists the levels and process parameters used in this study to machine AISI D3 steel.

**Figure 1:**
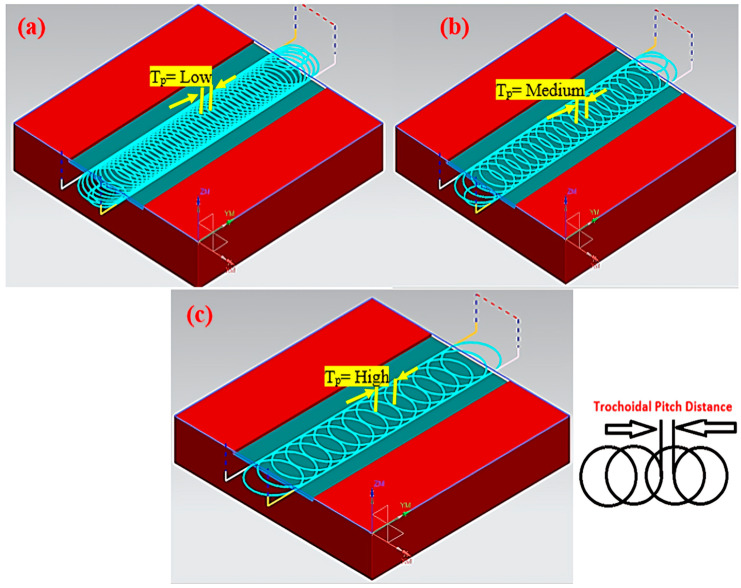
Trochoidal toolpath pitch distance simulation using Mastercam x6 under (**a**) low, (**b**) medium, and (**c**) high conditions.

**Figure 2:**
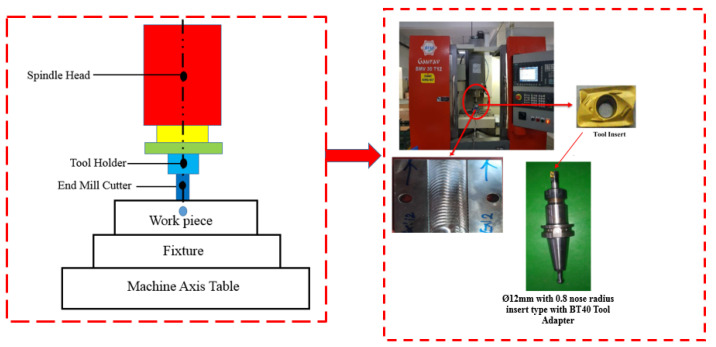
Details of the machining setup.

To perform end milling operations, tungsten carbide inserts (Sandvik Coromant, Sandviken, Sweden, ANSI R390-11 T3 08M-M) with a diameter of Ø12 mm and a corner nose radius of 0.8 mm, coated with solid titanium carbonitride, were selected. The tool was then mounted on the tool holder (ISO specification R390-012A16-11L) of the machine. A BT40 adapter was used for holding the tool. Trochoidal milling involves exerting forward motion and gradually milling the cutting tool with a series of continuous circles. The trochoidal machining module in the Mastercam X6 software tool (Mastercam UK Ltd., Manchester, UK) was utilized to simulate the trochoidal tool path, which is denoted in Figure 1. Here, the trochoidal pitch distance depends on the tool diameter. Therefore, to ensure the tool safety, the path pitch values were chosen to be less than 50% of the tool diameter, i.e., between 1.8 and 5.4 mm, and the loop diameter was determined using the CAM program based on the cavity. To machine the narrow slot, the cavity with a cross-section of 150 mm × 30 mm and a depth of cut of 1 mm per pass was selected. Figure 2 depicts the experimental setup utilized for this study.

### Measurement of Output Responses

An ACCRETECH (Tokyo Seimitsu Co., Ltd., Tokyo, Japan) make Surfcom 1400 G surface roughness tester made in Japan was used at the base of the milled surface to measure the roughness of every specimen using a 4 mm sample length and a 0.8 mm cut-off length. This instrument has several applications in industrial and research environments for assessing surface roughness, texture, topography, thin film characteristics, friction, and wear for ensuring product quality and performance. The following details pertain to the roughness measurement instrument. This tool is a contact stylus that has an arm length of 60 mm and a radius of 2 μm, which is equivalent to a 60° conical diamond tip size. The scan speed is 1.5 mm/s, and the measurement force is 0.75 mN. The detector can go up to 800 μm with a vertical resolution of 0.1 nanometers. The drive column may reach a vertical speed of 10 mm per second. The machine maintains an accuracy of 3 nm in the 0.2 mm vertical range and 15 nm in the 1 mm vertical range. The potential measurement error is within ±3 nm for measurements within the 0.2 mm vertical range and ±15 nm for measurements within the 1 mm vertical range. The end-milled surface was measured three times at various locations along the feed direction. Table 4 displays the results of the average surface roughness measurements. To obtain the cutting tool’s geometric features, 2D and 3D measurements were performed. To assess tool nose radius deformation after the milling operations, a Vision measuring system (VMS) was used.

**Figure 3:**
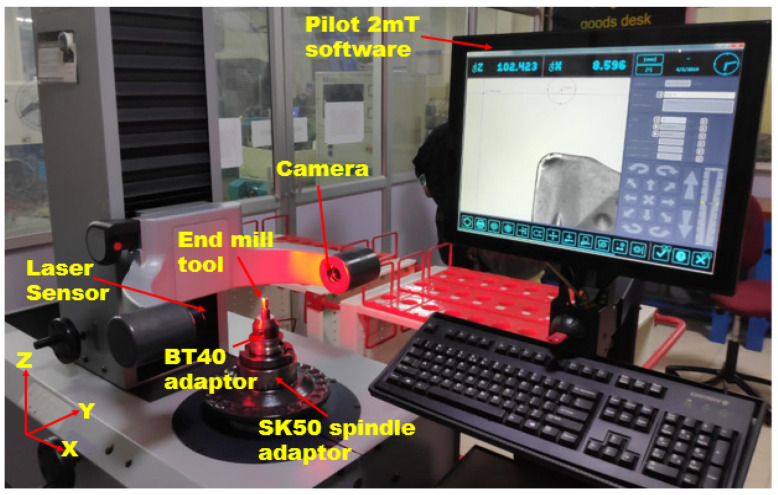
Measurement of the nose radius using the Zoller tool pre-setter.

Tool wear images and R_a_ were captured using a Vision Optive Lite OLM 3020 model built with VMS 3.1 software (Hexagon Manufacturing Intelligence, Uttar Pradesh, India). The model is equipped with a 1/3 inch high-resolution CCD camera that can take pictures with a pixel size as small as 1 μm. The magnification level can be adjusted from 30× to 180× using the camera’s built-in LED stage light and ring light. The results of calibrating the tool nose radius deviation using the Zoller Junior Plus V400 tool pre-setter (Zoller Inc., Deutschland, Germany) are shown in Figure 3. Sizes of up to 210 mm in length and 420 mm in width are possible for the pre-setter along the X axis. There is a precision of ±0.003 mm along the horizontal axis and an accuracy of ±0.005 mm along the vertical axis. The concentricity of the SK50 spindle is 0.002 mm, which gives it a reputation for outstanding accuracy. To retrieve attachment holds for tool calibration, it indexes the relevant data. Tools should not be longer than 320 mm or wider than 620 mm, and the table may hold up to 50 kg. The camera is a monochrome charge-coupled device (CCD) with a 7 × 6 mm chipset, which ensures precise tuning of the twelve red LEDs that comprise the lighting system.

**Figure 4:**
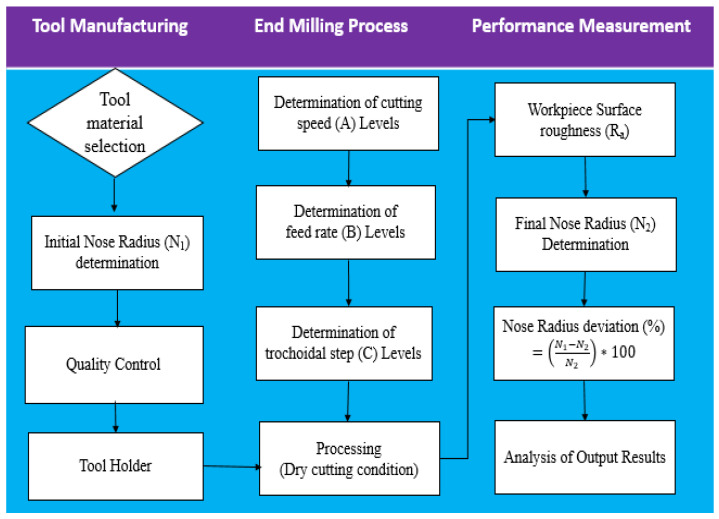
Experimental design approach used to determine the performance tool.

Before and after machining, Zoller tool pre-setter “Pilot 2mT” V400 software calibrated the nose radius variation value for the specified runs. The initial nose radius (*N*_1_) is 0.8 mm for every single tool. Equation (1) was used to compute the nose radius deviation:

(1) $$Nose\;Radius\;deviation,N\;\left(\%\right)=\left(\frac{N_{1}-N_{2}}{N_{2}}\right)\times 100$$

where, *N*_1_ and *N*_2_ denote the nose radius before and after the tool machining, respectively. Figure 4 displays the flow chart for the experimental design approach used to determine the performance tool.

## 3. Experimental RSM Design Matrix

RSM is an effective technique that is used in establishing the predictive models of the parameters that based on the experimental data. It is often used for optimization and developing mathematical models that explain the behavior of process elements and reactions. Based on the literature review, the systematic procedure for RSM consists of the following six steps. First, the dependent responses and the independent parameters are described. Thereafter, a design plan for the experiment is made using independent factors according to the face-centered CCD. This is followed by doing the right multiple regression analysis if, for instance, the variables are continuous [28]. The importance of factors and their combinations is then determined using ANOVA. Lastly, a confirmation test is done to verify the correctness of the model created. If it is correct, then a decision is made to accept the model. Otherwise, the model is rejected.

The factors of the present research include cutting speed, feed rate, and pitch distance, whereas the responses are surface roughness and nose radius. Table 4 presents the measured output values that are obtained. The first- and second-order mathematical models are found in Equations (2) and (3) and are derived from datasets [29,30]. These equations were used to build the empirical models, which explain the phenomenon within the data and give the best possible estimates.

(2) $$NX_{i}=d_{0}+d_{1}x_{1}+d_{2}x_{2}+\ldots \ldots +d_{n}x_{n}$$

(3) $$X_{i}=d_{0}+\overset{k}{\underset{i=1}{\sum }}d_{i}x_{i}+\overset{k}{\underset{i=1}{\sum }}d_{\mathrm{ii}}x_{i}^{2}+\overset{k}{\underset{i=1}{\sum }}\overset{k}{\underset{j=1_{i<j}}{\sum }}d_{\mathrm{ij}}x_{i}x_{j}\pm \epsilon$$

Here, the output responses are denoted as $X_{i}$, and the constant term is denoted as $d_{0}$. The coefficients of linear terms are represented by $d_{1},d_{2}\ldots \ldots .d_{n}$ in Equation (2). In addition, the linear, square, and interaction terms are $d_{i},\;d_{\mathrm{ii}},\;\mathrm{and}\;d_{\mathrm{ij}}$ in Equation (3). The input parameter $x_{i}$ represents the cutting speed (A), feed rate (B), and trochoidal pitch distance (C).

### 3.1. Developing Mathematical Relationships and Regression Analysis

The dynamics of the system are described by the regression model, a second-order polynomial quadratic model. The nonlinear Equation (3) can be solved by taking the variables’ logarithms and transforming them into a linear form so that regression models can be built using Design Expert software version 11. Based on the aforementioned, the response surface regression model’s empirical form was used to calculate the model’s coefficients.

It is important to mention that not all of the significant interaction factors have a significant impact on the milling performance. To determine the statistical difference between the parameters, ANOVA was used. The critical process factors obtained from ANOVA were then included in the final mathematical model relationships. The following relationships for the coded mathematical model are obtained using Equations (4) and (5).

R_a_ (µm) = 0.448 + 0.0172 × A + 0.0936 × B + 0.0447 × C + 0.005 × AB+ 0.004975 × AC + 0.0075 × BC + 0.0240 × A^2^ + 0.0140 × B^2^+ 0.0139 × C^2^ (4)

N (%) = 19.17 − 7.07 × A + 1.77 × B + 3.14 × C + 0.725 × AB −1.37 × AC + 0.0875 × BC + 1.37 × A^2^ + 0.9155 × B^2^ + 0.5905 × C^2^ (5)

### 3.2. Assessing the Accuracy of the Empirical Relationship

The empirical model performance was validated using ANOVA. These data are presented in Table 5 and Table 6 for surface roughness and nose radius deviation, respectively. The F-value measures the model’s significance. For the models presented in Table 5 and Table 6, the *p*-value is greater than F and less than 0.0001, which underlines the importance of the developed models [31]. Particularly, the interaction term (B × C) is significant for surface roughness, while (A × C) is significant for nose radius deviation. The second-order terms B^2^ and A^2^ were also significant for both surface roughness and nose radius deviation, respectively. The value of the lack of fit is low, which means that it is not significant.

The models possess a high prediction rate (R^2^) and acceptable levels of accuracy (AP). The surface roughness has a predicted R^2^ value of 0.9480 and an AP value of 21.203. Similarly, the nose radius deviation has a predicted R^2^ value of 0.9618 and an AP value of 20.61. This implies that the models are well-fit for predicting experimental results. Surface roughness and nose radius deviation have higher adjusted R^2^ values of 0.9405 and 0.9273, respectively. This shows that the created model is of greater significance. In addition, the coefficients of determination, R^2^ (predicted) and R^2^ (adjusted), are quite close. The coefficient of variation (Cv) is relatively low at 4.10 for surface roughness and 7.98 for nose radius deviation for the experiments, demonstrating that the experiments were done with high reliability and accuracy.

**Figure 5:**
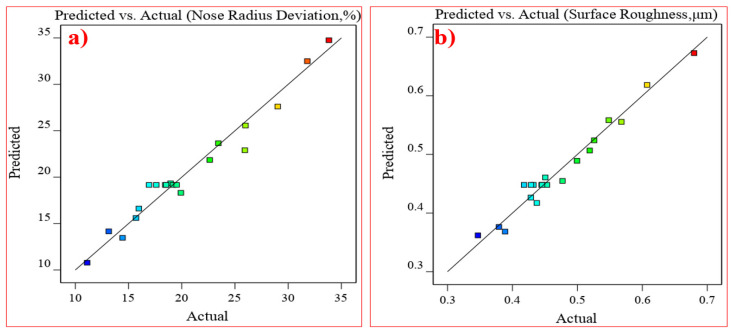
Prediction versus actual correlation for (**a**) nose radius deviation and (**b**) surface roughness.

Figure 5 displays the plot of observed and expected values, comparing the output response of AISI D3 tool steel specimens to the observed and predicted data of the empirical model derived from the analysis using correlation graphs. From the values of R^2^ obtained for the empirical models, the level of fit between the observed and the predicted values is quite high.

## 4. Results and Discussion

### 4.1. Influence of Trochoidal Parameters on Surface Roughness

**Figure 6:**
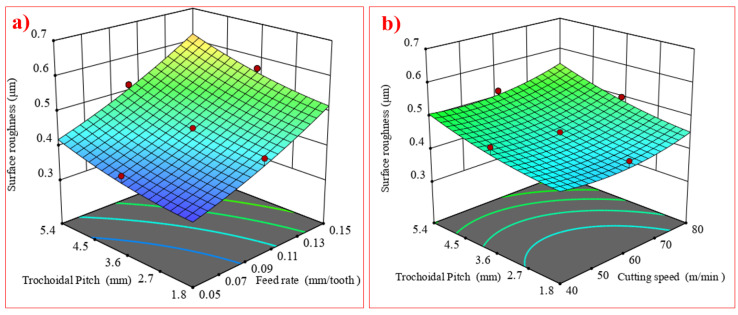
Interaction effects of (**a**) A and C on R_a_ as well as (**b**) B and C on R_a_.

Imperfections on a machined surface are difficult to remove after milling. The surface roughness (Ra) value is used to assess these imperfections. An efficient method for analyzing the behavior with two process variables is the 3D surface plot. In these 3D plots, the dependent response is put on the Z axis, while the independent factors are put on the X and Y axes [32]. The studies of various surface characteristics are presented in the form of surface plots. Figure 6a shows the mean R_a_ with cutting speed (A), feed rate (B), and trochoidal pitch distance (C). It was noted that the average surface roughness increases as the trochoidal pitch distance increases from 1.8 mm to 5.4 mm. Poor surface roughness results from the large, induced stress on the tool tip caused by the increased consecutive trochoidal pitch.

**Figure 7:**
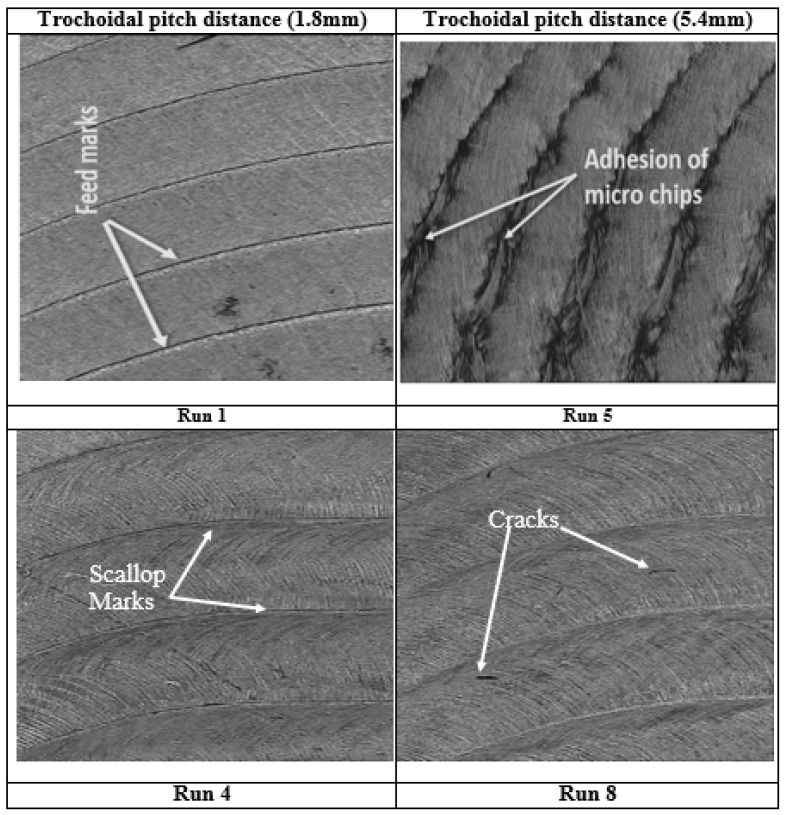
Influence of trochoidal pitch distance on surface texture.

The R_a_ value tends to decrease as the cutting speed is increased from 40 m/min to 80 m/min. This phenomenon is explained by the fact that when the cutting speed increases, there is a tendency for the friction between the workpiece and the cutting tool to diminish, which suppresses the creation of built-up edges [33]. Figure 6b shows that the surface roughness is exacerbated because of the high load on the tool, which increases the cutting force [11]. Figure 7 shows that the lace mark’s intensity, the workpiece’s side flow, the presence of cracks, and the adherence of chips on the machined surface all steadily increase.

### 4.2. Influence of Trochoidal Parameters on Tool Nose Radius Deviation

**Figure 8:**
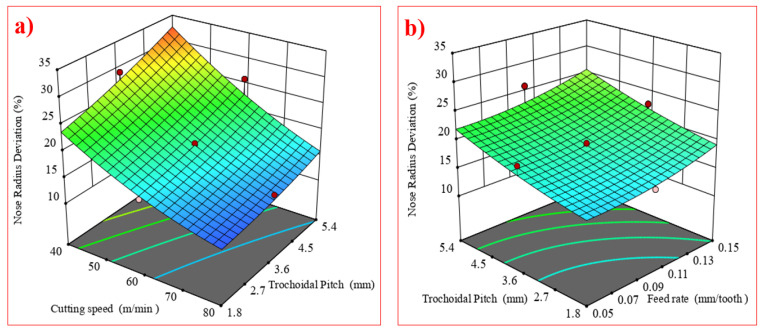
Interaction effects of (**a**) A and C on nose radius deviation as well as (**b**) B and C on nose radius deviation.

One of the most intricate and talked-about forms of wear is nose wear, which combines the characteristics of flank and crater wear at the cutting tool’s nose and flank. It is also categorized as a distinct type of wear around the corners of the tool, which is important for getting the greatest cutting performance. Figure 8a shows that when the cutting speed is increased from 40 m/min to 80 m/min, nose radius deviation is decreased because of less friction and less engagement of the tool. On the other hand, as the trochoidal pitch distance was increased from 1.8 mm to 5.4 mm, nose radius deviation tended to increase because of more engagement of the tool tip.

**Figure 9:**
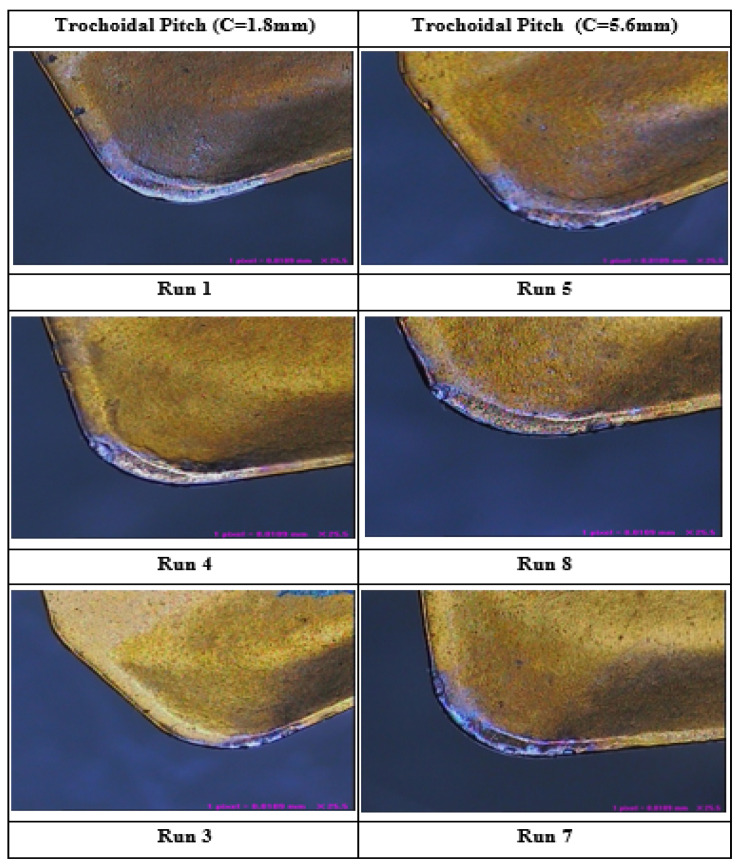
Tool deformation image captured using VMS (the run numbers are presented in Table 4).

Figure 8b indicates that the feed rate increases from 0.05 mm/tooth to 0.15 mm/tooth, and the nose radius deviation also increases. This notion is also supported by Figure 9, which shows that wear in the tool tips, especially on the cutting edge, leads to deformation. It also shows the trends in the irregularities of the tool tip, which causes the edge micro-geometry after milling to exhibit increased deviation from the initial nose radius. In general, the nose face is more prone to abrasion because of the frictional interactions with the workpiece and the movement of chips along the chip–tool contact zone, which leads to the formation of BUE on the cutting tool’s crater face [33].

### 4.3. Chip Morphology of Trochoidal Toolpath

**Figure 10:**
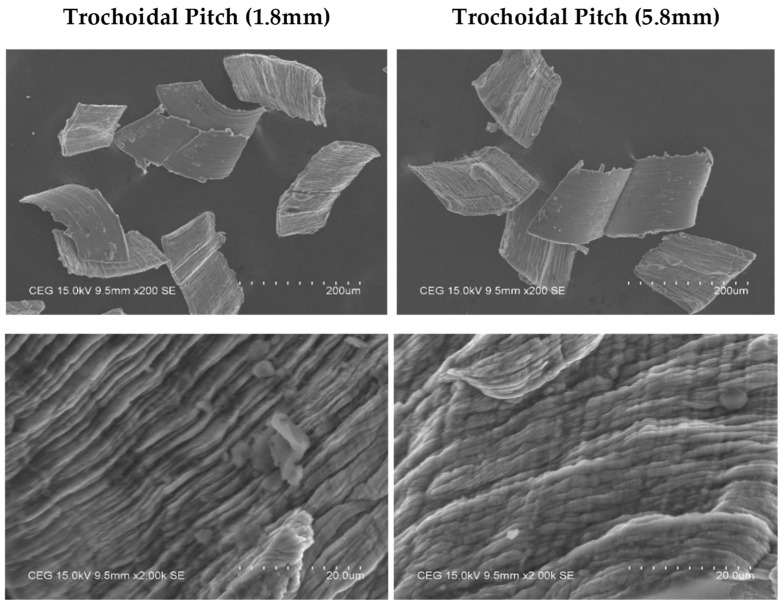
Chip morphology for trochoidal toolpath distances of 1.8 mm and 5.8 mm at A = 80 m/min and B = 0.05 mm/tooth.

Figure 10 displays the lamella structure for trochoidal pitch distances of 1.8 mm and 5.8 mm with cutting conditions of 80 m/min for A and 0.05 mm/tooth for B. The back surface of the chip in the lamella structure is characterized by a rough and jagged appearance, which is caused by a shearing action. It was observed that when the trochoidal pitch distance is low, there is a decrease in the engagement angle of the tool during the process. This decrease in angle leads to a significant reduction in the cutting load. Therefore, a uniform lamella structure is formed. Additionally, the chips were broken into tiny segmented pieces. The non-uniform lamella structure was observed at a trochoidal pitch distance of 5.8 mm. It is evident that significant shearing action occurs in the cutting zone. When the trochoidal pitch distance is increased, the tool tip experiences increased chatter and cutting forces, leading to high shear action on the material. Therefore, a significant amount of contact pressure and frictional force is encountered at the back surface as the chip glides across the tool rake face, leading to the formation of an uneven lamella structure.

### 4.4. Multi-Objective Optimization

**Figure 11:**
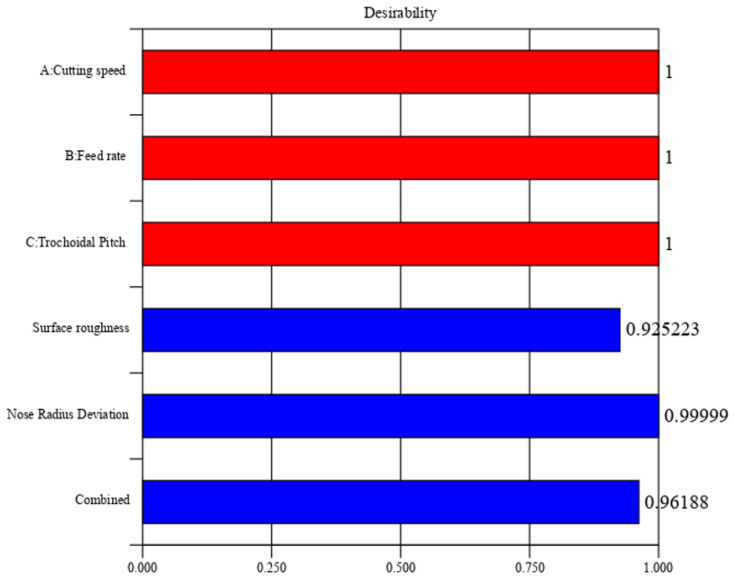
Plot of the 2D composite desirability histogram.

**Figure 12:**
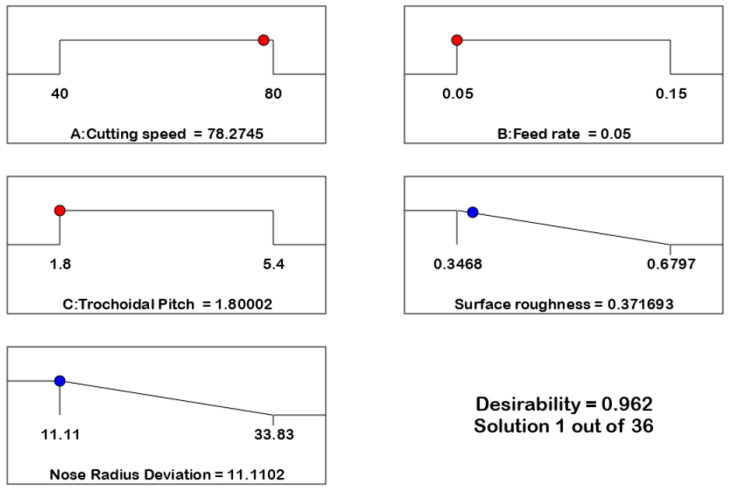
The numerical optimization ramp.

The desirability method is a widely used optimization strategy in industries for solving multiple objective problems. It aims to discover the optimal values for input variables. This approach was introduced by Derringer and Suich [28]. Desirability is a numerical value that varies from 0 to 1 and is determined by the level of closeness of the performance response to the desired outcome. Using Design Expert version 11, the desirability of this experiment was assessed. The R_a_ and nose radius deviation were optimized using the data obtained from the response surface methodology (RSM). Figure 11 displays the graphs of the numerical optimization, while Figure 12 shows the 2D composite desirability histogram distribution of the desirability. The ramp function assigns a desirability value to each component and output, as well as to the overall composite attractiveness. The input factor set or the output quality forecast for a certain quality feature is quantified in terms of the number of dots on ramps. The height of each dot corresponds to the level of acceptability of the output quality response, as specified in references [28,29,30].

The closest optimal area is determined to have a composite desirability value of 0.9618, which indicates the extent to which it meets the goal value. A multiple regression model was created to predict the output responses. The model was validated using experimental data and compared to the findings of confirmation trials. The confirmation tests were done three times using the optimal input factors. The optimal input variables for the confirmation studies were a cutting speed of 78 m/min, a feed rate of 0.05 mm/tooth, and a trochoidal pitch distance of 1.8 mm. Table 7 displays the results of the confirmatory test as well as the expected and actual values of the output responses based on the optimization technique.

Table 7 shows that the average values of the two performance responses of surface roughness (0.3717 μm) and nose radius deviation (11.11%) from the experimental results are very much in agreement with the RSM predicted values. and the error is quite small. These findings confirm that the developed models are significant within the range of the input factor levels.

### 4.5. The Outlook

One limitation of the study lies in the fact that the research focuses only on AISI D3 steel as the material of study. These findings, however, may not hold for other alloys that may exhibit different machining characteristics. Further, the experimental setup may involve lubrication or coolant procedures that are commonly used in the industrial environment. Although the present work determines the best parameters, more research on pitch distances may help to establish the weaknesses and strengths of trochoidal toolpath performance. Although there are other factors that can be considered when evaluating the machining performance, including cutting force and thermal issues, the key factors that are of interest in this study are surface quality and nose radius deviation.

From the findings of this study, important implications concerning the relationship between toolpath pitch distance and the surface finish and nose radius deviation when machining AISI D3 steel can be deduced. This study reveals that the use of trochoidal toolpaths has provided a significant enhancement in surface finish and minimized the nose radius deviation, and these factors are vital in the precision machining process. Thus, by adjusting the toolpath pitch distance, the overall surface finish can be improved, thus avoiding further operations like polishing or grinding. These optimal machining parameters can be particularly useful in industries that demand a high degree of accuracy, like aerospace and automotive industries, to guarantee that parts are manufactured to exact specifications.

In addition, the determination of the best operating conditions in machining AISI D3 steel can greatly minimize tool nose wear and increase tool durability. This is particularly relevant when tools are used in large numbers and are subject to frequent replacement, which leads to increased costs. The reduction in tool wear not only leads to lower material costs but also reduces the time taken to replace worn-out tools, thus increasing the overall production time. Optimal trochoidal toolpath parameters can be used to increase machining efficiency, and this is beneficial in the modern world where time is a key factor in manufacturing.

## 5. Conclusions

The present research work focuses on the analysis of the output responses of cutting speed, feed rate, and the trochoidal pitch distance during the end milling of AISI D3 cold work tool steel using a trochoidal tool path. Three input variables at three levels were investigated by conducting twenty trials; the CCD was used within the context of RSM. Second-order regression models were used to estimate the surface roughness and nose radius deviation. The following conclusions are drawn from this study:

1. The presented mathematical models for R_a_ and nose radius deviation are in good correlation with the experimental data obtained, with the difference between the experimental and calculated values ranging from 4.25% and 5.31%, respectively. High coefficients of determination (R^2^) are an indication of the model’s reliability.
2. From the F-ratio values, it is seen that feed rate and trochoidal pitch distance have a significant effect on the surface roughness, while all three input parameters affect nose radius deviation.
3. The intensity of the lace marks, microdamage on the tool, side flow of the workpiece, cracks, and chip adhesion on the machined surface reduce the surface finish of the workpiece.
4. Chip morphology studies revealed that an uneven lamella structure was obtained in the form of rough and jagged appearances when trochoidal pitch distance was increased.
5. The results from the vision measurement system regarding the tool nose radius deviation indicate that chipping, abrasion, and coating peel-off cause a decrease in the tool nose radius deviation. Larger deviations in tool nose radius, up to 33.83%, are noted when cutting speeds are low, the trochoidal steps are low, and the feed rates are high.
6. The desirability-based multi-objective optimization technique determined that the optimal process parameter setting is 78 m/min for A, 0.05 mm/tooth for B, and 1.8 mm for C. The data suggest that increasing the cutting speed, drop-in feed rate and trochoidal pitch value improves the quality attributes of the output.

This study offers useful information to researchers and the machine tool industry for selecting optimal parameter settings to obtain the desired surface finish and nose radius deviation using the trochoidal toolpath. The use of these optimal parameters improves the quality of machined parts, hence reducing the costs of tools and increasing the efficiency of machining.

## Figures and Tables

**Table 1:** The weight percentage of the chemical components of AISI D3 cold work tool steel.

Element	Vanadium (V)	Manganese (Mn)	Nickel (Ni)	Silicon (Si)	Carbon (C)	Chromium (Cr)	Iron (Fe)
wt.% content	0.25	0.4	0.31	0.3	2.1	11.5	Balance

**Table 2:** Details on the mechanical properties of cold work tool steel AISI D3.

Billet Materials	Mechanical Properties of AISI D3 Steel				
	Density (kg/cm^3^)	Hardness (HRC)	Heat Conductivity (W/mK)	Yield Strength (N/mm^2^)	Tensile Strength (N/mm^2^)
AISI D3	7.7	55–64	20	850	970

**Table 3:** The coded values of process variables.

Variables	Symbol	Units	Coded Values		
			(−1)	(0)	(+1)
Cutting speed	A	m/min	40	60	80
Feed rate	B	mm/tooth	0.05	0.1	0.15
Trochoidal pitch	C	mm	1.8	3.6	5.4

**Table 4:** Test results of the proposed experiments.

Run	Input Factors			Output Performance		
	A (m/min)	B (mm/tooth)	C (mm)	Surface Roughness (Ra) (µm)	Nose Radius (N_2_) (mm)	Nose Radius Deviation (%)
1	40	0.05	1.8	0.3468	1.045	23.44
2	80	0.05	1.8	0.3792	0.9	11.11
3	40	0.15	1.8	0.5259	1.081	25.99
4	80	0.15	1.8	0.5483	0.949	15.70
5	40	0.05	5.4	0.4283	1.173	31.80
6	80	0.05	5.4	0.4506	0.921	13.14
7	40	0.15	5.4	0.6074	1.209	33.83
8	80	0.15	5.4	0.6797	0.987	18.95
9	40	0.1	3.6	0.4771	1.127	29.02
10	80	0.1	3.6	0.4994	0.935	14.44
11	60	0.05	3.6	0.3887	0.999	19.92
12	60	0.15	3.6	0.5678	1.034	22.63
13	60	0.1	1.8	0.4375	0.952	15.97
14	60	0.1	5.4	0.519	1.08	25.93
15	60	0.1	3.6	0.4449	0.963	16.93
16	60	0.1	3.6	0.4324	0.99	19.19
17	60	0.1	3.6	0.4532	0.981	18.45
18	60	0.1	3.6	0.4286	0.971	17.61
19	60	0.1	3.6	0.4178	0.982	18.53
20	60	0.1	3.6	0.4456	0.994	19.52

**Table 5:** ANOVA for surface roughness.

Source	SS	d.f.	MS	F-Value	*p*-Value Prob > F	Remarks
Model	0.1213	9	0.0135	34.38	<0.0001	significant
A	0.0029	1	0.0029	7.52	<0.0001	
B	0.0875	1	0.0875	223.18	<0.0001	
C	0.0200	1	0.0200	51.02	<0.0001	
A × B	0.0002	1	0.0002	10.510	<0.0001	
A × C	0.0002	1	0.0002	0.5049	0.4936	
B × C	0.0004	1	0.0125	25.15	<0.0001	
A^2^	0.0016	1	0.0016	4.03	0.0726	
B^2^	0.0005	1	0.0095	14.37	<0.0001	
C^2^	0.0005	1	0.0005	1.37	0.2694	
Residual	0.0039	10	0.0004			
Lack of fit	0.0031	5	0.0006	3.56	0.0947	not significant
Pure error	0.0009	5	0.0002			
Total	0.1252	19				
Standard Dev.	0.0198			R^2^	0.9480	
Mean	0.4739			Adj R^2^	0.9405	
Cv %	4.10			Pred R^2^	0.8152	
				Adeq Precision	21.203	

**Table 6:** ANOVA for nose radius deviation.

Source	SS	d.f.	MS	F-Value	*p*-Value Prob > F	Remarks
Model	680.71	9	75.63	27.94	<0.0001	significant
A	500.41	1	500.41	184.86	<0.0001	
B	31.29	1	31.29	20.56	<0.0001	
C	98.85	1	98.85	36.52	<0.0001	
A × B	4.23	1	4.23	1.56	0.2395	
A × C	14.91	1	14.91	10.51	<0.0001	
B × C	0.0612	1	0.0612	0.0226	0.8834	
A^2^	5.16	1	15.16	9.91	<0.0001	
B^2^	2.30	1	2.30	0.8514	0.3779	
C^2^	0.9588	1	0.9588	0.3542	0.5650	
Residual	27.07	10	2.71			
Lack of fit	22.39	5	4.48	4.79	0.0554	not significant
Pure error	4.68	5	0.9356			
Total	707.78	19				
Standard Dev.	1.65			R^2^	0.9618	
Mean	20.61			Adj R^2^	0.9273	
Cv %	7.98			Pre R^2^	0.8037	
				Adeq Precision	20.6071	

**Table 7:** Confirmatory optimum factor level and output performance.

**Description**	Input Parameters			$R_{a}\;\left(\mu m\right)$		Nose Radius Deviation (%)	Error (%)
	A (m/min)	B (mm/tooth)	C (mm)	Value	%[Error]	Values	
Desirability optimal solution	78	0.05	1.8	0.3717	4.25	11.11	5.31
Optimum (Actual)	78	0.05	1.8	0.3559	-	10.52	-

## Data Availability

The original contributions presented in the study are included in the article. Further inquiries should be directed to the corresponding author.

## Abbreviations

Notation	
AISI	American Iron & Steel Institute
ANOVA	Analysis of variance
HRC	Hardness measured with the Rockwell test for hard materials
CAM	Computer-aided manufacturing
RSM	Response surface methodology
CCD	Central composite design
MRR	Material removal rate
SS	Sum of squares
MS	Mean square
VMS	Vision measuring system
d.f.	Degree of freedom
Symbol	
C	The trochoidal step refers to the distance between the centers of adjacent paths or revolutions (mm)
A	Cutting speed (m/min)
B	Feed rate (mm/tooth)
R_a_	Surface roughness (μm)
X_i_	Estimated output response
x_i_	Input parameter
d_0_	Free term of the regression equation
d_i_	Coefficients of linear terms
d_ij_	Coefficients of square terms
∈	Experimental error
d_1_, d_2_,…d_n_	Coefficients of linear terms
X_1_	Before machining the tool
X_2_	After machining the tool
